# A Case Report on an Uncommon Presentation of Giant Cell Tumor of the Tendon Sheath in the Infrapatellar Region

**DOI:** 10.7759/cureus.80918

**Published:** 2025-03-20

**Authors:** Arın Celayir, Hasan Marangoz, Gamze Göktürk Özcan, Nuraddin Abdullaev, Vedat Burkay Camurdan, Bedri Karaismailoglu

**Affiliations:** 1 Department of Orthopedics and Traumatology, Istanbul University-Cerrahpasa, Cerrahpasa Faculty of Medicine, Istanbul, TUR; 2 Department of Pathology, Henry Ford Hospital, Detroit, USA; 3 Department of Orthopedics and Traumatology, Kilis State Hospital, Gaziantep, TUR; 4 Department of Orthopedics and Traumatology, Taksim Training and Research Hospital, Istanbul, TUR

**Keywords:** excisional biopsy, giant cell tumor of the tendon sheath, infrapatellar region, localized nodular tenosynovitis, tenosynovial giant cell tumor

## Abstract

Giant cell tumor of the tendon sheath (GCT-TS) is a benign yet locally aggressive soft tissue neoplasm that typically arises in synovium-lined structures, including tendon sheaths, bursae, and joints. Also known as localized nodular tenosynovitis, this tumor primarily affects the hands and fingers but can occur in other anatomical locations. GCT-TS is histologically characterized by multinucleated giant cells within a background of mononuclear stromal cells.

While it is generally nonmetastatic, the tumor carries a risk of local recurrence following surgical removal. Recognizing the clinical presentation, histopathological features, and appropriate management strategies is essential for effective treatment and recurrence prevention.

Here, we present the case of a 34-year-old woman with a one-year history of pain in the infrapatellar region. Imaging revealed a well-defined soft tissue mass posterior to the patellar tendon, which was surgically excised. Histopathological examination confirmed the diagnosis of nodular-type GCT-TS. The patient's symptoms resolved completely postoperatively, and no recurrence was observed during a five-year follow-up period, highlighting the effectiveness of surgical excision in managing this rare tumor location.

## Introduction

Giant cell tumor of the tendon sheath (GCT-TS) is a benign lesion of uncertain etiology, with proposed contributing factors including inflammation, trauma, toxins, allergies, clonal chromosomal abnormalities, and aneuploidy [1,2]. The condition was first described by Jaffe et al. in 1941 [3]. Approximately 85% of GCT-TS cases occur in the fingers, while 12% are in the knee, elbow, hip, or ankle [4]. Although GCT-TS can occur at any age, it most commonly presents between 30 and 50 years of age, with a 2:1 female predominance [5]. GCT-TS in the knee is extremely rare, and the typical tumor size is relatively small, averaging 2 cm [5]. Reports in the literature of cases involving male patients, younger age groups, tumors located in the knee, or larger tumor sizes remain limited. While antecedent trauma has been suggested as a potential trigger, only 15% of cases have a definite history of trauma [6].

GCT-TS is characterized by the presence of multinucleated giant cells dispersed within a fibrous or histiocytic mononuclear stromal background. While commonly affecting the hands and fingers, its occurrence in the infrapatellar region is rare. These giant cells, often resembling osteoclasts, are a hallmark of the tumor. The stromal cells vary in morphology, displaying either spindle-shaped or epithelioid characteristics. Additionally, hemosiderin deposition is commonly observed, contributing to the lesion's characteristic appearance. Macroscopically, GCT-TS typically presents as a nodular, well-defined mass. Microscopically, it is composed of a mixture of mononuclear stromal cells, multinucleated giant cells, foam cells, and hemosiderin-laden macrophages, with variable cellularity and occasional mitotic figures.

Treatment primarily consists of careful local excision, with a preference for microscopic excision to minimize recurrence risk. In some cases, postoperative radiotherapy may be considered. Despite surgical removal, recurrence remains a concern. One of the key clinical challenges is the nonspecific nature of GCT-TS symptoms, which may lead to diagnostic delays or misdiagnosis, especially in atypical locations such as the infrapatellar region. In this case, the initial clinical suspicion did not favor GCT-TS; however, following excisional biopsy, histopathologic examination confirmed the diagnosis. This case highlights the diagnostic challenge of GCT-TS in unusual anatomical sites and underscores the importance of considering it in the differential diagnosis of unexplained knee pain and localized swelling. By emphasizing the role of imaging and histopathologic confirmation, this report contributes to the existing literature on the recognition and management of GCT-TS in rare locations, reinforcing the need for clinicians to maintain a high index of suspicion to ensure timely and appropriate treatment.

## Case presentation

A 34-year-old woman presented with a one-year history of pain in the left knee's infrapatellar region. She had a prior history of left knee arthroscopy. Informed consent was obtained from the patient before any procedure was performed.

**Figure 1:**
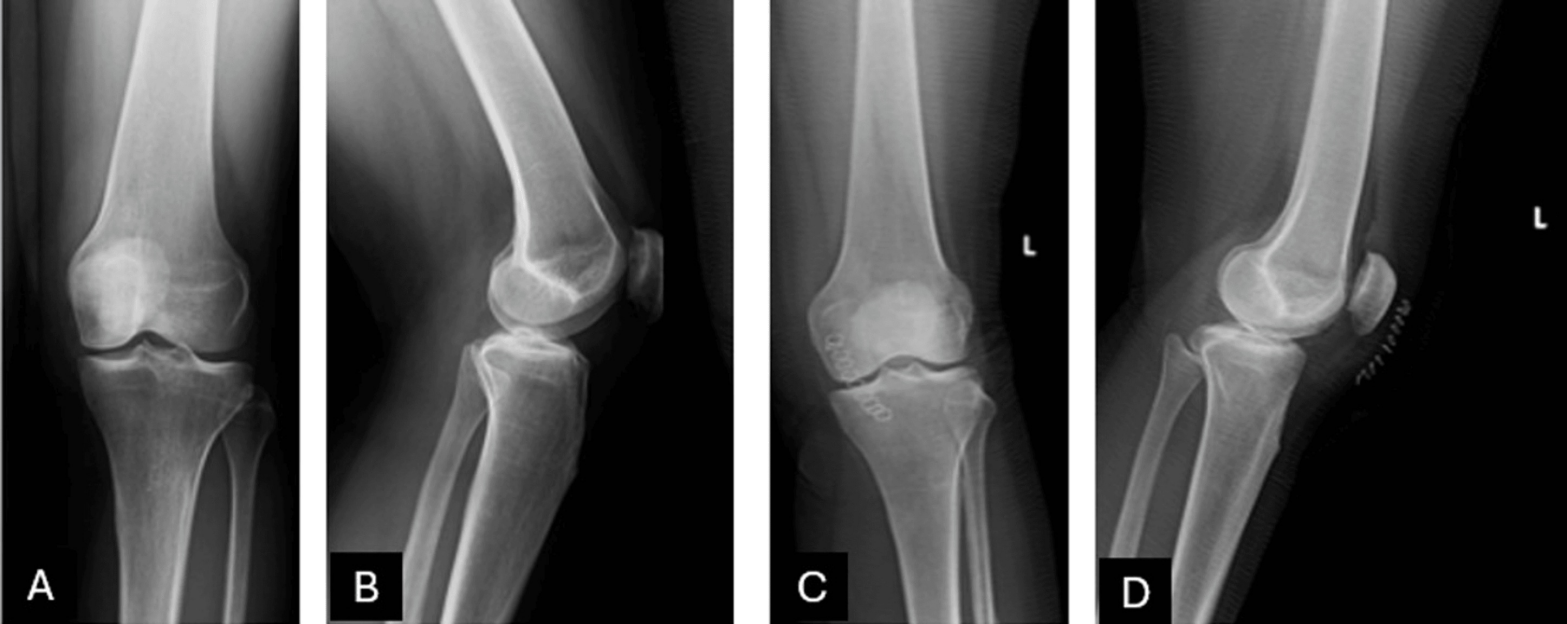
Preoperative and early postoperative radiological images of the patient. (A) Preoperative anteroposterior knee X-ray. (B) Preoperative lateral knee X-ray. (C) Early postoperative anteroposterior knee X-ray. (D) Early postoperative lateral knee X-ray. No significant osseous pathology is observed

On examination, the patient had a normal and painless range of motion with no signs of instability. Minimal swelling and localized tenderness were noted in the infrapatellar region. There were no systemic symptoms such as night sweats, fever, or weight loss. Additionally, no lymph node enlargement was observed, and blood test results were within normal limits. Direct radiography did not reveal any obvious bone pathology (Figure 1).

**Figure 2:**
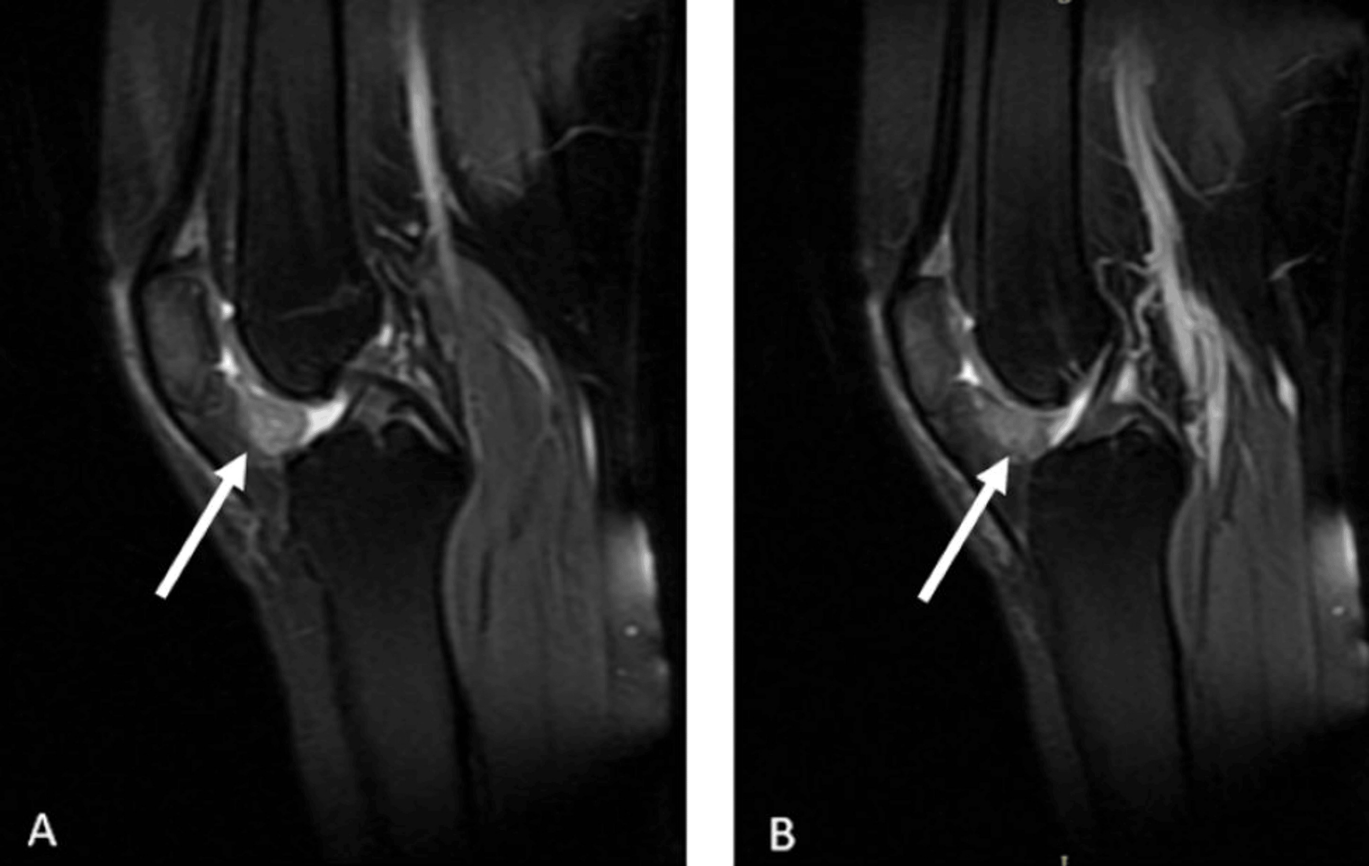
The patient's T2-weighted sagittal MRI sections. (A) The lesion is clearly visible within the affected region, demonstrating hyperintense characteristics. (B) Another sagittal section, showing the extent and morphology of the lesion The white arrows indicate the lesion MRI: magnetic resonance imaging

**Figure 3:**
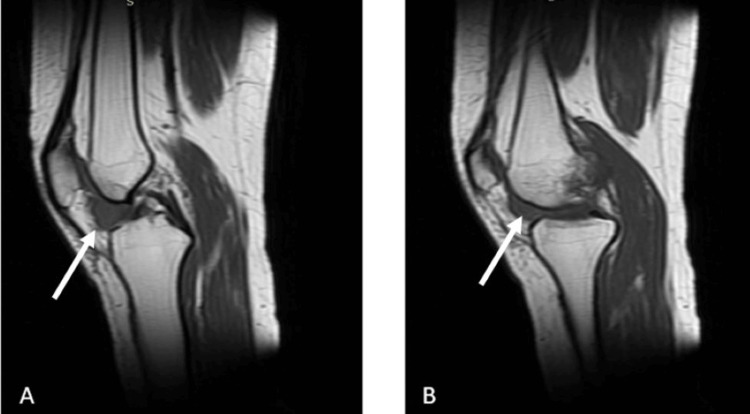
The patient's T1-weighted sagittal MRI sections. (A) The lesion appears as a hypointense area within the affected region, indicating its distinct contrast against the surrounding normal tissue. (B) Another sagittal section displaying the lesion’s extent and internal characteristics, further delineating its borders and involvement The white arrows indicate the lesion MRI: magnetic resonance imaging

**Figure 4:**
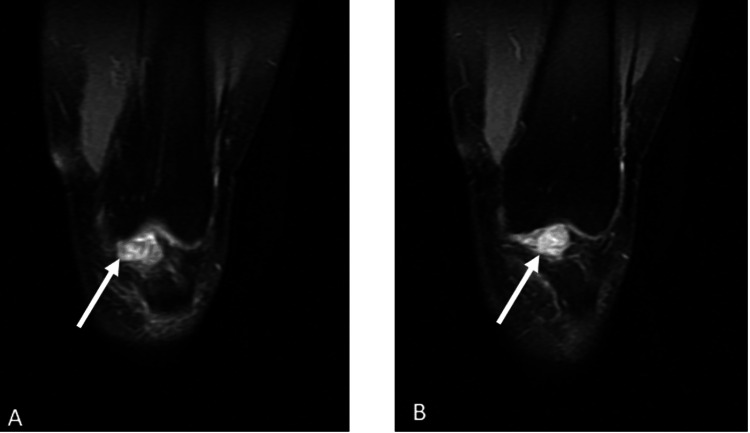
The patient's T2-weighted coronal MRI sections. (A) The lesion is visible as a hyperintense area within the affected region, showing its spatial relationship with adjacent anatomical structures. (B) Another coronal section further illustrating the lesion’s extent and orientation, with increased signal intensity highlighting its contrast against surrounding tissues The white arrows indicate the lesion MRI: magnetic resonance imaging

**Figure 5:**
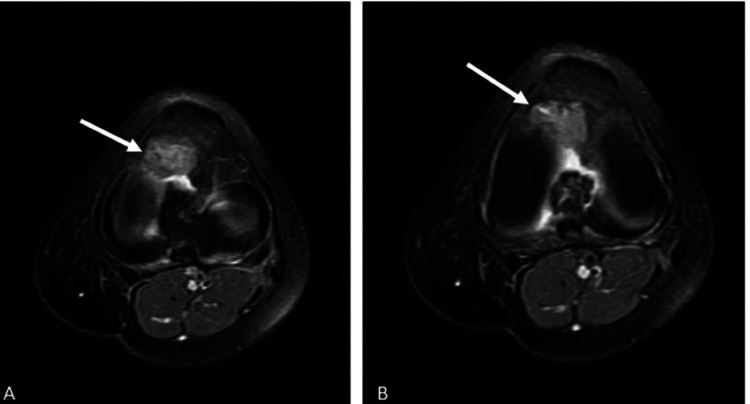
The patient's T2-weighted axial MRI sections. (A) The lesion appears as a hyperintense area in the axial plane, demonstrating its shape and extent relative to surrounding structures. (B) Another axial section further delineating the lesion’s borders and internal characteristics, providing additional details on its spatial orientation The white arrows indicate the lesion MRI: magnetic resonance imaging

Magnetic resonance imaging (MRI) revealed a well-defined soft tissue mass in the infrapatellar region posterior to the patellar tendon (Figures 2-5).

**Figure 6:**
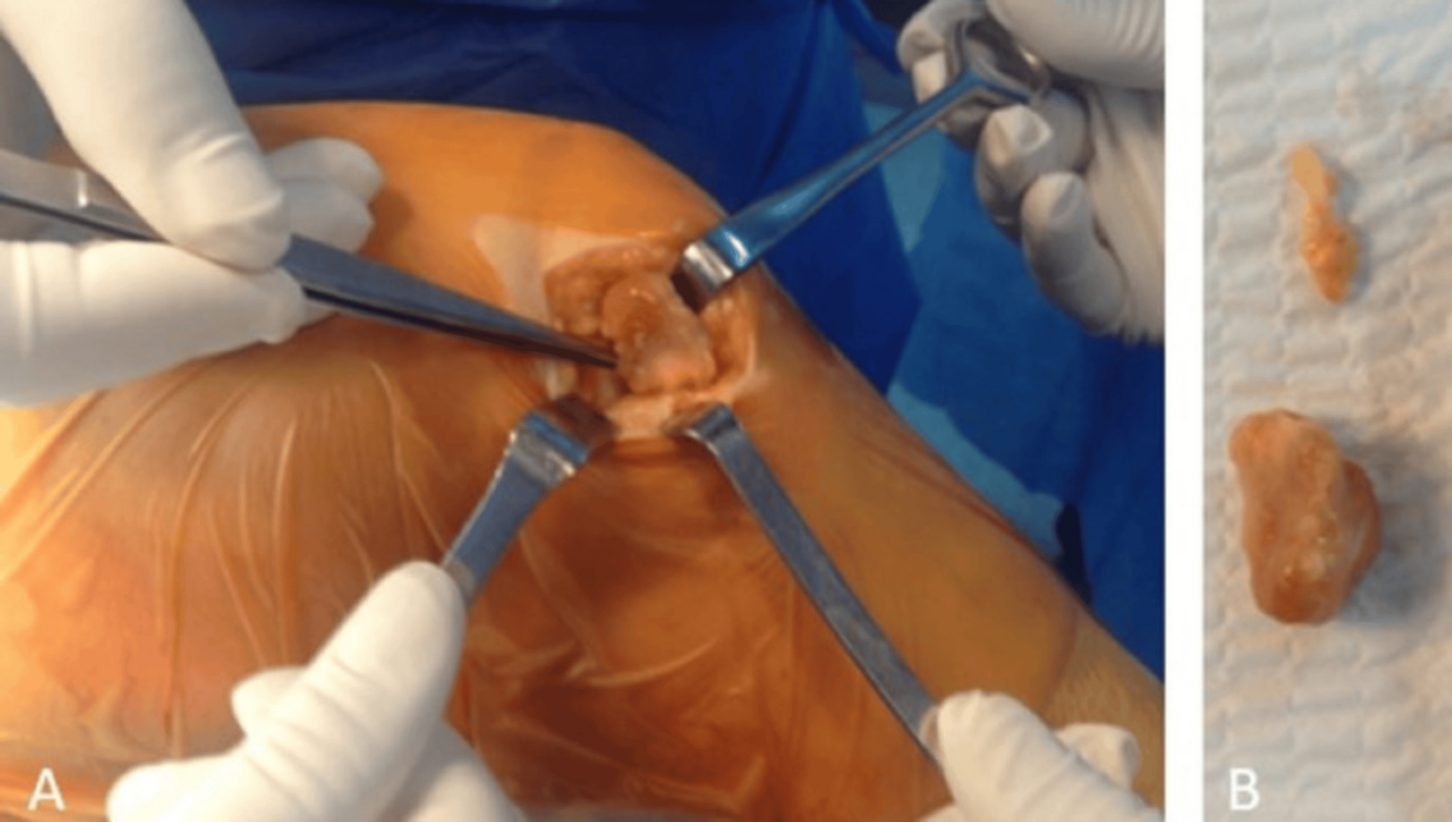
Intraoperative images of the patient. (A) Lesion being removed from the knee joint. (B) Removed lesion from the knee joint

The mass was surgically excised, and an excisional biopsy was performed. A lobulated mass measuring 4 × 2.5 × 1.5 cm was obtained, attached to the joint capsule by a stalk (Figure 6).

**Figure 7:**
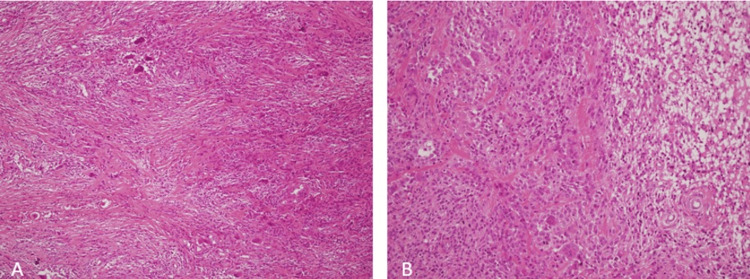
Nodular-type tenosynovial giant cell tumor composed of giant cells, mononuclear cells, and xanthomatous cells. (A) H&E 100×. (B) H&E 200× H&E: hematoxylin and eosin

Histopathological examination confirmed the diagnosis of nodular-type GCT-TS, characterized by a proliferation of mononuclear stromal cells interspersed with multinucleated giant cells, foam cells, and hemosiderin-laden macrophages. The tumor exhibited variable cellularity, occasional mitotic figures, and a well-demarcated, lobulated architecture without signs of malignancy (Figure 7).

The patient's symptoms resolved after surgery, and no recurrence was observed during the five years of follow-up. Postoperatively, she underwent a structured rehabilitation program that included pain management, gradual weight-bearing, and strengthening exercises to restore knee function. Regular clinical and imaging follow-ups were conducted to monitor for recurrence, with no signs of tumor regrowth or functional impairment.

## Discussion

Imaging plays a pivotal role in the evaluation and diagnosis of GCT-TS. Radiographs are often the initial imaging modality, revealing soft tissue masses and potential erosion of adjacent bone. However, MRI is particularly valuable in providing detailed information regarding tumor extent and characteristics. GCT-TS typically appears hypointense on T1-weighted images and hyperintense on T2-weighted images, aiding in differentiation from surrounding tissues. Additionally, MRI helps assess adjacent structure involvement and guides surgical planning. Ultrasound may also be utilized, offering real-time imaging and assisting in identifying cystic components within the tumor.

In our case, a preliminary diagnosis was made using MRI, leading to the decision to proceed with surgical treatment. The tumor was excised en bloc with clear margins, adhering to best practices to minimize recurrence risk. Care was taken to preserve surrounding structures, including the patellar tendon and synovium, ensuring optimal functional recovery. Histopathologic examination of intraoperative specimens confirmed the diagnosis of GCT-TS. The chosen surgical approach aligns with established recommendations, emphasizing complete excision while preserving joint function and reducing recurrence rates.

GCT-TS originates in the synovial lining of tendon sheaths and predominantly affects the hands and fingers. It primarily involves the distal extremities, with a predilection for the flexor tendons of the fingers and wrist. Although less common, it can also occur in other joints, such as the knees, ankles, and feet. Our patient presented with a rare localization of GCT-TS in the knee. Following MRI imaging and biopsy, the diagnosis was confirmed. Despite its infrequent occurrence in the knee, clinicians should remain vigilant for such cases [6].

The primary treatment for GCT-TS is surgical excision, aiming for complete tumor removal due to its locally aggressive nature and potential for recurrence. Surgery is performed to relieve symptoms and minimize the risk of local relapse. Although GCT-TS is generally benign and nonmetastatic, recurrence can occur postoperatively. In cases where complete excision is challenging due to tumor location or size, adjuvant therapies such as radiotherapy or intralesional steroid injections may be considered to reduce the recurrence risk [7]. In the five-year postoperative follow-up of our patient, no signs of recurrence were detected, and the patient remained asymptomatic. Routine check-ups indicated a return to normal daily activities.

GCT-TS, tenosynovial giant cell tumor, and pigmented villonodular synovitis (PVNS) are histologically and clinically similar tumors commonly found in synovial joints and tendon sheaths. Wang et al. highlighted that GCT-TS can be mistaken for PVNS [8]. This distinction is crucial during differential diagnosis [9].

## Conclusions

The rare occurrence of GCT-TS in our patient’s knee, coupled with its unusually large size, emphasizes the need to consider this condition in the differential diagnosis of knee masses. Our findings highlight the limitations of imaging modalities like MRI, which, while essential for preliminary assessment, cannot replace the diagnostic accuracy of histopathological examination following excisional biopsy.

This case underscores the importance of timely recognition and accurate diagnosis of GCT-TS to prevent misdiagnosis and ensure appropriate treatment, particularly in patients presenting with nonspecific knee symptoms. For clinicians managing similar cases, a high index of suspicion is necessary when evaluating localized knee masses, especially those with atypical presentations. Complete surgical excision with clear margins remains the cornerstone of treatment to minimize recurrence risk and preserve joint function.
